# Miscellaneous

**Published:** 1875-09

**Authors:** 


### Miscellaneous.

### ETHER AND CHLOROFORM AS ANÆSTHETICS.

The "Boston notion" that ether is to be preferred to chloroform as the safer agent for anæsthetical purposes is confirmed by a recent communication made to the *Societa Medico-fisica*, of Florence, by Professor Schiff, abstracts of which we find in recent foreign journals. The paper embodies the results of more than five thousand experiments made by its author in order to ascertain the differences in the anæsthesia produeed by these two agents. In the following points the two anæsthetics agree: they produce paralysis of conscious sensation, paralysis of the movement of the voluntary muscles, paralysis of respiration, of circulation, and, finally, of the heart and vasomotor nerves. The chief differences occur in the order in which the paralysis takes place. With ether, respiratory paralysis takes place while circulatoin and blood pressure remain within the limits compatible with life. In other words, respiratory paralysis precedes vascular paralysis in the case of ether, so that the vascular pressure remains sufficiently high, directly after respiration has ceased, to enable artificial respiration to be resorted to with success ; or, in the word of the professor, "when some automatic respiratory movements have been obtained, it may be taken as certain that respiration will continue, and that the animal will live." With chloroform, on the contrary, vascular paralysis frequently comes on first, so that an amount of this agent which would be insufficient to produce respiratory paralysis will often bring on vascular paralysis, accompanied by such a lowering in the blood pressure as to render artificial respiration of no avail, as no interchange takes place between the gases of the air and those of the blood. Hence although automatic respiratory movements may be induced, life is not recalled, and respiration again ceases when artificial aid is removed. In the present state of our knowledge, it may therefore be said that the surgeon is responsible for the death of an individual by etherization, while he is not so if chloroform has been used. Therefore chloroform should be rejected, and only ether used.

### A COMMON CAUSE OF APOPLEXY.

In an able article on apoplexy, in the *Popular Science Monthly*, Dr. J. R. Black, gives the following hint to brain-workers :

"A middle-aged physician said one day to the writer : 'As I was walking down the street after dinner I felt a shock in the back of my head, as if some one had struck me ; I have not felt well since. I fear I shall die, just as all my ancestors have, of paralysis. What shall I do ?' The answer was, 'Diminish the tension on the blood-vessels, and there need be no fear of tearing them in a weak place.' Now this expresses in plain terms the exact cause of apoplexy in the great majority of instances; and it is one, too, which every one has it in his power to prevent. A blood-vessel of the brain has lost some of its elastic strength; food is plenty, digestion is good; blood is made in abundance, but little is worked off by exercise; the the tension on every artery and vein is at a maximum rate; the even, circuitous flow is temporarily impeded at some point, throwing a dangerous pressure on another; the vessel which has lost its elastic strength gives way, blood is poured out, a clot is formed which, by its pressure on the brain, produces complete unconsciousness. This is the apoplectic stroke. It will be perceived that there are two leading conditions upon which the production of the stroke depends—a lessened strength in the vessel, and an increased tension on it."

### THE DIAGNOSIS OF THE "LEAD LINE."

In chronic lead poisoning a blue line appears on the gums. But this may be simulated by other substances deposited there. Dr. Gras recommends that when we are in doubt whether a given blue-line on the gum be due to lead or not, we should excise a fragment of the gum containing the line, with a fine sharp scalpel or the point of a lancet, wash it with a camel's hair pencil, and add a drop of glycerine; if necessary, flatten it out with needles, and examine it under the microscope with a low power. If the line be due to lead, in the midst of the normal tissues of the gum we shall find capillaries injected, filled and obstructed by blackish granules. These capillaries are in loops, or semicircular, or like double hooks, the outlines varying somewhat according to the section. In very old lead-lines the capillary walls are less evident, and their outlines some what indistinct. If a piece of buccal mucous membrane be excised, we should use carmine with glycerine, and a little dilute acetic acid, which shows the mucous papillæ, and the capillary network. He suggests that in fatal lead-colic, the intestinal capillaries and the nerves of the solar plexus should be examined in the same way for lead. It has long since been proposed to examine the lead-line by a simple microscope, or in other words, a one or two inch bi-convex lens; when, if in the capillaries, as the true lead-line is, it will be seen clearly to be dotted, and to follow the course of the vessels.

### CARBONIC OXIDE IN TOBACCO SMOKE.

Dr. Otto Krause, in *Dingler's Polytechnic Journal*, states that he finds a considerable quantity of carbonic oxide constantly present in tobacco smoke, and that the after-effects of smoking are prinicipally caused by this poisonous gas, as the smoker never can prevent a part of the smoke from descending to the lungs, and thus the poisoning is unavoidable. He is of opinion that the after-effects are all the more energetic, the more inexperienced the smoker is, and he thus explains the unpleasant results of the first attempts at smoking, which are generally ascribed to nicotine alone.

### THE GOAL OF MEDICAL SCIENCE.

The Editor of the London *Medical Press and Circular* thus sums up the future aims of medicine:

To make our fellow citizens enjoy a healthy life, and prevent the enormous waste of infantile life which invariably goes on in our large towns, ought to be the medical religion of the remaining quarter of this remarkable century. To live a long life, and to live all our lives, ought by this time to be perceived to be the practical teaching of all medical science; and no other consideration should allow these wholesome truths to be obscured from the view of the medical profession.

### BIOGRAPHY.

Dr. M. McCarty, of Pulaski, Tenn., was born Oct. 26, 1832, in Clark Co., Ky., and was educated at Bethel College.

In the year 1864, he moved from Russellville where he then resided to Toronto, Canada, where he studied dentistry with Dr. Elliott. He returned to Russellville in the Spring of 1865, and continued the study of the profession with Dr. Jones of that city.

On the 26th of Oct., 1865, he was married to Miss A. P. Stewart and afterward, during the years 1866 and 1867, he attended one course of lectures at the Cincinnati Dental College.

In May 1867, Dr. McCarty moved to Pulaski, Tenn., where he practiced his profession until Jan. 1st, 1874, when he returned to Russellville where he died in the following March.

Dr. McCarty was a member of the Tenn. Dental Association. He was a quiet unassuming gentleman, honest and thorough in all his professional work, and but for his short life and feeble health he would have attained a high order of excellence in his profession.

S. J. Cobb.
Chair. Com. on Mem. Tenn. Dental Asso.

Died in Ashtabula, Ohio, August 24th, 1874, Dr. G. W. Nelson, aged forty years.

Expecting each month to see the above announcement in the Register, I have refrained from calling your attention to the sad fact that our profession has lost one of its tried and truest members.

Although modest and retiring to a marked degree, Dr. Nelson was efficient, skilful, industrious and ambitious to excel. Above all, he was thoroughly honest and conscientious, deserving and possessing the confidence of co-laborers in his chosen profession, and of the community where he resided. Knowing him well, as a friend, and as a professional brother, I faintly express my love for him by this small tribute to his sterling worth. M.

### ERRATA.

In the article on Dental Education, contributed by Dr. L. G. Noel, to our June No., there occurred some typographic errors which, but for the tinge of the ludricrous one of them presents, would doubtless have called down a fiat anathema upon the head of our unfortunate compositor. On page 253, 11th line from the bottom, strike out "the" before whiners.

On page 255, where the writer speaks of medicine as the "grand old mother" of dentistry, the types insist upon making her the "old grand mother."

Page 258, 13th line from the bottom, for medical read dental.

